# Pathophysiology of Fever and Application of Infrared Thermography (IRT) in the Detection of Sick Domestic Animals: Recent Advances

**DOI:** 10.3390/ani11082316

**Published:** 2021-08-05

**Authors:** Daniel Mota-Rojas, Dehua Wang, Cristiane Gonçalves Titto, Jocelyn Gómez-Prado, Verónica Carvajal-de la Fuente, Marcelo Ghezzi, Luciano Boscato-Funes, Hugo Barrios-García, Fabiola Torres-Bernal, Alejandro Casas-Alvarado, Julio Martínez-Burnes

**Affiliations:** 1 Neurophysiology, Behavior and Animal Welfare Assessment, Unidad Xochimilco, Universidad Autónoma Metropolitana, Mexico City 04960, Mexico; jocelyn.gomez.ilp@gmail.com (J.G.-P.); luciano.boscato@gmail.com (L.B.-F.); fabitorber19@gmail.com (F.T.-B.); ale0164g@hotmail.com (A.C.-A.); 2 School of Life Sciences, Shandong University, Qingdao 266237, China; dehuawang@sdu.edu.cn; 3 Laboratório de Biometeorologia e Etologia, FZEA-USP, Faculdade de Zootecnia e Engenharia de Alimentos, Universidade de São Paulo, Pirassununga 13635-900, Brazil; crisgtitto@usp.br; 4 Animal Health Group, Facultad de Medicina Veterinaria y Zootecnia, Universidad Autónoma de Tamaulipas, Ciudad Victoria 87000, Mexico; vcarvajal@docentes.uat.edu.mx (V.C.-d.l.F.); hbarrios@docentes.uat.edu.mx (H.B.-G.); 5 Animal Welfare Area, Faculty of Veterinary Sciences (FCV), Universidad Nacional del Centro de la Provincia de Buenos Aires (UNCPBA), Buenos Aires 7000, Argentina; ghezzi@vet.unicen.edu.ar

**Keywords:** febrile response, hyperthermia, infectious process, pathogen, pyrogen, thermoregulation, thermogenesis, thermolysis, thermoregulatory behavior, animal welfare

## Abstract

**Simple Summary:**

The current immune, metabolic, and neural pathways and the structures involved in developing the febrile response and, in return, to thermal homeostasis are known. There is still much information to be revealed about the underlying mechanisms that participate in the febrile process and the control of energy balance. This review analyzes the recent advances in pathophysiological mechanisms of the febrile process, the heat loss in an animal with fever, thermoregulation, the adverse effects of fever, and recent scientific findings related to different pathologies in farm animals through the use of infrared thermography, a fast, reliable, and non-invasive tool that is useful in the early detection of pathologies of clinical importance.

**Abstract:**

Body-temperature elevations are multifactorial in origin and classified as hyperthermia as a rise in temperature due to alterations in the thermoregulation mechanism; the body loses the ability to control or regulate body temperature. In contrast, fever is a controlled state, since the body adjusts its stable temperature range to increase body temperature without losing the thermoregulation capacity. Fever refers to an acute phase response that confers a survival benefit on the body, raising core body temperature during infection or systemic inflammation processes to reduce the survival and proliferation of infectious pathogens by altering temperature, restriction of essential nutrients, and the activation of an immune reaction. However, once the infection resolves, the febrile response must be tightly regulated to avoid excessive tissue damage. During fever, neurological, endocrine, immunological, and metabolic changes occur that cause an increase in the stable temperature range, which allows the core body temperature to be considerably increased to stop the invasion of the offending agent and restrict the damage to the organism. There are different metabolic mechanisms of thermoregulation in the febrile response at the central and peripheral levels and cellular events. In response to cold or heat, the brain triggers thermoregulatory responses to coping with changes in body temperature, including autonomic effectors, such as thermogenesis, vasodilation, sweating, and behavioral mechanisms, that trigger flexible, goal-oriented actions, such as seeking heat or cold, nest building, and postural extension. Infrared thermography (IRT) has proven to be a reliable method for the early detection of pathologies affecting animal health and welfare that represent economic losses for farmers. However, the standardization of protocols for IRT use is still needed. Together with the complete understanding of the physiological and behavioral responses involved in the febrile process, it is possible to have timely solutions to serious problem situations. For this reason, the present review aims to analyze the new findings in pathophysiological mechanisms of the febrile process, the heat-loss mechanisms in an animal with fever, thermoregulation, the adverse effects of fever, and recent scientific findings related to different pathologies in farm animals through the use of IRT.

## Methodology

**Figure 1:**
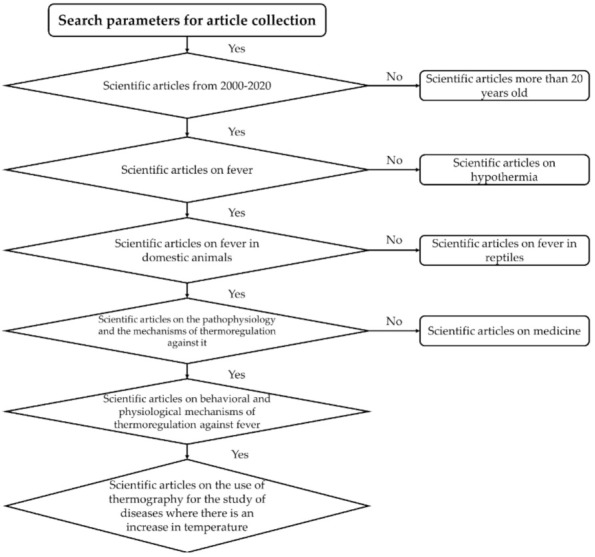
Flowchart showing the criteria used during the search of scientific articles for the review.

For our study topic, the literature published between 2000 and 2020 was retrieved from ScienceDirect, PubMed, and Web of Science. The search was subsequently refined for physiological changes during fever, mechanisms of thermoregulation in domestic animals, and recent studies using infrared thermography to understand those responses. The authors also reviewed the sources cited in the articles identified to broaden the search and add relevant information (Figure 1).

## 1. Introduction

Fever is a cardinal symptom that occurs when homeostasis is altered due to the presence of infectious or non-infectious stimuli, which is why it is recognized as a hallmark of disease [1,2,3].

Regarding fever, a wide variety of questions have arisen to define whether it is a mechanism that should be treated or not, and their answers are based on the findings obtained at the neurophysiological level, related to the hypothalamic control of fever and the development of thermoregulatory responses [4]. Despite this, some still question whether their study helps measure pathologies, in spite of the fact that scientific evidence indicates that it can be used for their detection [2,4]. However, given the scarcity of quantitative, studies of non-invasive tools that allow us to understand and interpret the pathophysiological processes of fever in farm animals are still valid [5,6].

Concerning this issue, infrared thermography (IRT) has proven to be a reliable auxiliary method that allows for the early detection of pathologies that, in addition to affecting animal health and welfare, represent economic losses for farmers [7]. However, the standardization of the protocols for the use of this tool is still needed, so that, together with the complete understanding of the physiological and behavioral responses involved in the febrile process, it is likely to have timely solutions to serious problem situations [7,8].

For this reason, the present review aims to describe and analyze the pathophysiological mechanisms of the febrile process, the mechanisms of heat loss in an animal with fever, and recent scientific findings when studying farm animals with infrared thermography.

## 2. Pathophysiology of Fever and Adverse Effects

### 2.1. Pathophysiology of Fever

Fever is considered an acute phase response generated by the body when it undergoes an infection or systemic inflammation; it is a regulated mechanism that is present from primitive organisms. This defense strategy can be observed in vertebrates or homeothermic organisms and invertebrates or ectotherms [9]. It is known that the central nervous system (CNS) receives all necessary information to know the organism state at any time and therefore is informed of the existing damage in the periphery. This information is sent through a sensory system and reaffirmed with the arrival of immune chemical signals to the CNS. Likewise, upon receiving these signals, the body activates defense strategies, such as fever [10].

It is essential to mention that the hypothalamus is considered the biological thermostat of the organism and has stipulated stable temperature ranges, according to each species, which keeps the heat loss and production adjusted [11]. During fever, neurological, endocrine, immunological, and metabolic changes occur that cause an increase in the stable temperature range, allowing the core body temperature to rise significantly to stop the invasion of the offending agent and restrict the damage to the organism [10].

When a foreign agent enters the host, it is considered an exogenous pyrogen. It is a primary inducer of fever that enters from the external environment and produces endogenous pyrogens that function as messengers that emit the signal to the biological thermostat to activate the defense strategy. Various stimuli produce fever, but mainly by the presence of exogenous pyrogens, such as microorganisms (viruses, bacteria, fungi, and among others), inflammatory agents, synthetic products, substances derived from destroyed tissue, complement fragments, and specific metabolites [12]. In general, exogenous pyrogens are structurally constituted by molecules that function as a physical barrier against antimicrobial agents and pathogens associated with molecular pathogens (PAMPs) that allow the body to detect the entry of this foreign agent. An example of PAMP is lipopolysaccharides (LPSs), which are the main components of the outer membrane of Gram-negative bacteria [12,13].

When the organism is challenged with the entry of an exogenous pyrogen, in the first place, interactions of molecular structures of the exogenous pyrogen are generated with molecular structures of the cells in charge of immunity. An example of these interactions is the binding of LPS with glycoproteins called lipopolysaccharide-binding proteins (LBPs) and CD14 glycoproteins, which are present in antigen-presenting cells [10,12]. The binding or interaction allows the molecular weight of LPSs to be reduced so that they can bind to specific receptors on the cell surface of immune cells called Toll-like receptors (TLRs) [14]. When stimulated by low-molecular-weight LPS, these receptors initiate a signaling cascade to activate the transcription of genes that encode the production of endogenous pyrogens, also known as pyrogenic cytokines.

Currently, interleukin 1 (IL-1 α and β), tumor necrosis factor-alpha (TNFa), interleukin 6 (IL-6), and interferon-gamma (IFN-g) are recognized as the main pyrogenic or pyrogenic endogenous cytokines; however, interleukin 8 (IL-8) and interferon-alpha (IFN-a), likewise, have been reported to have the pyrogenic potential [15]. It is not easy to describe all the pyrogenic cytokines functions, since they are highly interrelated with other chemical substances that act synergistically. Sometimes, only the action of a cytokine is necessary to trigger a series of chemical reactions [12,15]. Most of these molecules are glycoproteins that have other relevant effects on the system, since they are secreted into the general circulation by cells of the immune system, such as macrophages, lymphocytes, monocytes, B cells, dendritic cells, and mast cells, located in strategic points of the organism [3].

It is known that exogenous pyrogens can stimulate endogenous pyrogen production or act directly on the circumventricular organs of the brain. Suppose that pyrogenic cytokines mediate the stimulation; in that case, the organism is responsible for transporting them through the general circulation to the vascular organ of the terminal lamina (OVLT), which is one of the seven predominant cellular structures in the anterior hypothalamus within the terminal lamina located in the optic recess of the anteroventral end of the third ventricle [16]. The circumventricular organs, such as the OVLT, are highly vascular, and the blood–brain barrier (BBB) in these areas is reduced; therefore, it can be directly stimulated by pyrogenic substances [11].

Scientific evidence confirms that, when endogenous pyrogens stimulate OVLT, prostaglandin E2 (PGE2) production pathways are activated, involving enzymes such as phospholipase A2 (PLA 2), Cyclooxygenase (COX 2), and PGE Synthetase [17]. It is known that PLA2 is the enzyme that allows the release of arachidonic acid, which is the precursor of prostaglandin, which, through enzymatic reactions, obtains this precursor from the phospholipids present in the membrane of local microglial cells. When arachidonic acid is released, the COX 2 enzyme catalyzes a reaction on this substance to obtain prostaglandin G2 as a product that, finally, through an enzymatic conversion mediated by PGE synthetase, the final product, PGE2, is obtained [12,18]. The synthesis of prostaglandin E2 can also be carried out in Kupffer cells, located in the liver, in response to stimulation by LPS present in Gram-negative bacteria. Likewise, various studies describe that exogenous pyrogens are transported by the bloodstream mainly to the liver, where, when they come into contact with phagocytic Kupffer cells, the release of both endogenous pyrogens and PGE2 is stimulated [19].

PGE2 is reported as the most abundant lipid component during an infection or inflammation, and due to its lipid nature, it can cross the blood–brain barrier (BBB) of the areas where circumventricular organs, such as the OVLT, are located [20]. PGE2 is considered the last febrile mediator, interacting directly with specific G-protein-coupled receptors located in the cerebral endothelium situated in OVLT. As a result of the interaction with these receptors, signals are transmitted that reach the parvocellular portion of the paraventricular nucleus (HPV) and the ventromedial preoptic nucleus (VMPO) of the brain [11,21].

It is important to note that, with the arrival of these signals, mechanisms are originated to improve the release of neurotransmitters and alter the reference range of thermoregulation, which causes an increase in temperature until fever occurs [11]. Structures such as HPV, the pituitary, some endocrine organs, and the brain stem produce fever effector mechanisms that activate sympathetic portions of the medulla responsible for stimulating brown adipose tissue, producing chills, and increasing blood pressure through vasoconstriction, which are mechanisms that aim to generate heat [11]. Moreover, because the body changes its reference value, indicating a higher value than the previous one, the organism presents signs similar to hypothermia; in other words, during this change, the body detects the previously normal temperature as a decreased temperature by the increase in the reference value, and this sign disappears until the body equals the temperature to the new stipulated value [9].

In the most recent investigations, the existence of an alternative pathway that has joint action with humoral transport or through cytokines has been speculated. This alternative is called the neural pathway that generates fever through peripheral signals transported by branches of sensory nerves and vagus nerves associated with the anterior preoptic hypothalamic area (POA) [10]. Several studies confirmed this speculation since, when performing vagotomies, specifically of the hepatic nerve branches, they suppressed or attenuated the febrile response stimulated by administering different doses of pyrogenic substances. Currently, it has been described that the stimulation of these vegetative branches is fundamentally the work of exogenous pyrogens that circulate in the vessels supplying the abdominal cavity [22].

In summary, with an external pyrogen agent, mechanisms have triggered that cause the production of endogenous pyrogens in the body. When these substances are secreted into the general circulation by the immune cells that produce them, they travel through the circulation until they reach organs, where they provoke the synthesis of PGE2 that has the role of stimulating the release of neurotransmitters to alter the reference value of thermoregulation and finally generate mechanisms that raise the core temperature until triggering a defense strategy called fever [10,11,12].

### 2.2. Adverse Effects of Fever

In general, the effects or complications of fever are observed in organisms exposed to higher temperatures and for extended periods [11].

The increase in temperature to generate fever has various effects ranging from direct cell damage to irreversible systemic and neurological effects. From a local or molecular point of view, a significant temperature rise is required to cause denaturation of proteins and, as a chain effect, destabilize the cell membrane, altering its transport capacity until causing cell death, which, in most cases, is programmed by the same cell or called apoptosis [16,23]. In the same way, by denaturing proteins, fundamental processes, such as DNA and RNA replication or synthesis, are interrupted. That is why cells in mitosis are more heat-sensitive than cells in any other stage. It is known that RNA synthesis can be restored once the fever ceases, compared to DNA replication, which, although the defense strategy is stopped or decreased, remains suspended for a more extended period, resulting in damage to the cell structure and function [23].

Concerning systemic complications, prolonged exposure to elevated temperature induces alterations in the permeability of the digestive tract that allow the passage of microorganisms, mainly bacteria, to extraintestinal sites, such as blood and mesenteric lymph nodes [16]. This process called intestinal bacterial translocation corrupts the integrity of the gastrointestinal barrier to induce the presence of toxins that act as stimuli to secrete cytokines with inflammatory potential; likewise, due to the damage to the gastrointestinal structure and cell death, the presence of free radicals increases, and these free radicals participate by evoking oxidative stress that synergistically contributes by increasing cytotoxicity to cell death [24].

Studies describe that, when macroscopically inspecting the bodies of rodents exposed to heat, glomerular capillary dilation, stasis in blood vessels of various sizes, and reduction of vascular spaces were observed after exposure to fever for prolonged periods, thus reaffirming the theory that, during the increase in temperature, blood flow and glomerular filtration are reduced [25]. Furthermore, it has been described that the cause may be related to the action of the renin–angiotensin hormones that decrease the blood volumes in the renal tract until causing hypoperfusion that finally triggers lesions in the renal epithelium [26].

Scientific evidence confirms that the dilation of blood vessels of various sizes in conjunction with stasis, extravasation, and hemorrhages are vascular lesions commonly observed in various systems in response to prolonged exposure to elevated temperatures [16]. An example is hypotension in the cardiac circulation due to these lesions in the myocardial vessels. In addition, there is an extravasation of the red blood cells that leads to vascular stasis until causing the separation of structures such as myocardial fibers. Likewise, in studies with rodents, dilation of the pulmonary capillaries and intra-alveolar hemorrhages were observed. Similarly, the liver presents organic functional failure, since, in response to the defense strategy, it dilates the centrilobular veins and the vessels of the portal area until generating stasis and hemorrhages, which are also related to the serum levels of some enzymes of hepatic origin [25]. As with mechanisms with intimate connections, liver damage is considered responsible for the alteration in the homeostatic system by inducing mechanisms such as transient thrombocytopenia, platelet inhibition, and prolonged clotting times, since the correlation between increased serum levels of enzymes of liver origin such as alanine aminotransferase (ALT) with coagulopathies during temperature elevations has been observed [27].

One of the systems that suffer the most remarkable alterations due to fever is the neurological one. At elevated temperatures, the ranges depend on the species; the hypothalamus loses the ability to coordinate temperature regulation, compromising various brain structures to the point of causing multi-organ dysfunction and irreversible brain damage [28]. In addition, the existence of a relationship between increased temperature and ischemic infarcts that exacerbate late neuronal death of cortical cells, cerebellar granular cells, and the dorsal root ganglion has been reported, causing neurological deficit [29]. Likewise, it is known that, at high temperatures, cellular swelling originates due to the alteration in cell membrane transport and neuronal death that becomes noticeable shortly after the end of prolonged fever [30].

In ancient descriptions, the radical damage caused by fever during pregnancy is mentioned, since the stages of embryonic development, specifically neuronal migration and proliferation, are considered susceptible to changes in temperature [31]. Maternal infections have been suggested to alter the fetal environment drastically since the secretion of pro-inflammatory cytokines is stimulated to increase their serum levels, and as a result, there is abnormal brain development and impairs memory on the offspring [11,32]. A recent study in pregnant guinea pigs exposed to high temperatures demonstrated the association of embryonic death, caused by abnormal cardiac development, with exposure to heat during neuronal migration stages [28]. It is also suggested that autism is a sequel to infections during pregnancy due to the abrupt interruption of brain development. However, it has also been related to the suspension of fever [11].

### 2.3. Difference between Fever and Hyperthermia

It is essential to understand the difference between a hyperthermic state and a febrile one and the changes between the structures involved in each mechanism. Moreover, the pathways that trigger the presence of each state. At present, it is known that body temperature elevations are multifactorial in origin and are called differently [33].

Hyperthermia is described as a rise in temperature due to alterations in the thermoregulation mechanism. In other words, the body loses the ability to control or regulate body temperature during this state. In comparison, fever is a controlled state, since the body adjusts its stable temperature range to increase body temperature without losing the thermoregulation capacity [34]. Based on this, it is known that, in the case of fever, the body is organized to trigger processes that generate heat from energy intending to reach or equal the new reference value [35]. The body detects the presence of an exogenous pyrogen and, through molecular mechanisms, produces endogenous pyrogens directed to areas of the hypothalamus to stimulate the catalysis of other agents responsible for facilitating the release of neurotransmitters to change the reference value. Once the value increases, the body responds by causing the activation of thermogenesis in brown adipose tissue (BAT) and skeletal muscle [16]. In contrast, when the organism suffers from a hyperthermic state, the response is directed to diminish or dissipate the heat to avoid injuries or organic failures. Therefore, in general, the nervous system perceives the alterations through heat-sensitive neurons located in strategic points and generates a stimulus that travels through various nervous structures to reach blood vessels and glandular structures, which, when stimulated, originate mechanisms to dissipate heat [6,36,37].

The etiology of each state is another marked difference. Hyperthermia is generated mainly by the increase in heat production with the simultaneous decrease of the mechanisms that favor heat loss; and fever can be caused by pathogens, inflammatory processes, or any alteration in organic functioning that causes the body to generate a defense strategy to try to stop or restrict the damage [33].

**Figure 2:**
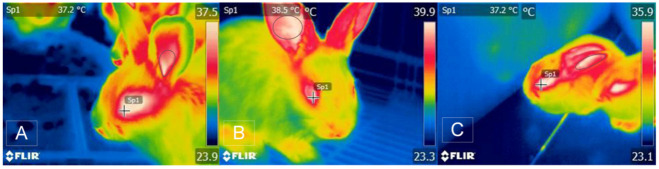
Rabbit thermograms before, during, and after being subjected to a transport simulation model. (**A**) The specimen is observed before being subjected to vibratory stimulation with a temperature of 37.2 °C in the lacrimal caruncle and a surface temperature of more than 37 °C in the periocular region and part of the pinna (indicated with an oval). (**B**) The specimen is subjected to a vibratory stimulus for 10 min, at an intensity of 4 Hz, with the presence of a 1.3 °C higher temperature in the lacrimal caruncle and increase of approximately 2.9 °C by 70% of the surface of the pinna (indicated with an oval). (**C**) The specimen is seen in the recovery phase, 20 min after having been subjected to the transport simulation model, at again 37.2 °C in the lacrimal caruncle and a temperature of the ear 4 °C lower than in thermogram (**B**). Comparing the thermograms, it is concluded that the rabbit presented a transient and monophasic hyperthermic response due to the effect of the stress generated through the stimulus of vibrations, since its temperature gradually decreases until it returns to its basal state once the stimulus has ended. Images obtained with an FLIR E 50 camera equipped with an 18 mm FOL lens at a resolution of 240 × 180 pixels, (emissivity = 0.95, distance = 0.5 m).

Recent studies have shown that acute psychological stress elevates body temperature values and speculates that regions such as the medial amygdala and the prefrontal cortex are involved by exerting negative feedback on the POA to activate the functioning of the hypothalamic–medullary–sympathetic axis (HMS). However, the communication routes between these areas are not well clarified [38]. In various species, it has been found that psychological hyperthermia can be caused by exposing them to stressful factors; in these animals, an elevation of the core temperature (CT) of 2 °C was shown in less than 30 min, which demonstrated the transitory increase of the CT [39,40]. For example, in rabbits subjected to transport stress simulation, psychological hyperthermia occurs (see Figure 2). Despite this, current reports mention the possibility that chronic stress is considered a febrile state instead of hyperthermic, since it was reported that, during repeated and prolonged exposure to a stressor, the production and secretion of proinflammatory cytokines are generated by stimulation of microglia; however, the need for more studies to confirm this theory is evident [41].

## 3. How Does an Animal with a Fever Thermoregulate? (Neurophysiological Responses to Temperature Control)

Thermoregulation is essential to optimize the immune response by mediating resistance mechanisms and inducing the mobilization of energy reserves towards defense mechanisms during infectious processes [42]. The immune, metabolic, and neuronal systems play an essential role in modifying the thermoregulation set point and body temperature [43].

Changes in body temperature can make the niche for infectious pathogens less hospitable by altering the environment according to its thermal preference and restricting access to essential nutrients for its proliferation and survival. Therefore, thermoregulatory mechanisms that increase and decrease body temperature are adaptive strategies of the host to promote tolerance defenses and confer a survival advantage by reducing infectious potential [42].

In addition to generating resistance mechanisms towards pathogens, the host must reach thermal homeostasis in charge of physiological and neuronal circuits through BAT thermogenesis, thermogenesis of musculoskeletal tissue with tremors or chills, hemodynamic changes, activation of evaporative pathways, and heat-seeking or cold-seeking behaviors related to the disease [43].

### 3.1. Central Modulation of Fever

The IL-6–COX2–PGE2 axis modulates the febrile process. During infection, the induction and maintenance of fever involve coordinating the immune system and neural circuits within the central and peripheral nervous systems. Immune detection of infection begins with the binding of PAMPs to pathogen recognition receptors (PRRs), such as Toll-like receptors (TLRs), which are expressed by populations of immune cells, including macrophages, neutrophils, and dendritic cells (DCs) [42].

PGE2 produced by vascular endothelial cells in the brain is considered an essential pyrogenic mediator of fever [44]. This lipid effector molecule integrates afferent signals from pyrogenic cytokines produced in response to pathogenic stimuli with efferent signals involving neurotransmitters that increase core body temperature. PGE2 is also synthesized in the periphery before the detection of circulating cytokines. It is produced by hematopoietic cells after LPS-mediated activation of TLR4 and travels across the blood–brain barrier to initiate fever. LPS-induced fever occurs through autonomous mechanisms driven by the binding of PGE2 to PGE2 receptor 3 (EP3) expressed by thermoregulatory neurons in the preoptic region of the hypothalamus (POA), a feverish and thermoregulatory center within the brain [45]. Endotherms raise body temperature by releasing norepinephrine, which increases thermogenesis in brown adipose tissue and induces vasoconstriction in the extremities to reduce passive heat loss. Furthermore, signaling through the neurotransmitter acetylcholine stimulates the musculature to convert stored chemical energy into thermal energy and increases global metabolic rates [42].

LPS-induced TLR4 signaling stimulates the synthesis of pyrogenic cytokines IL-1, IL-6, and TNF at the site of infection and in the brain. In particular, multiple cell types in the brain (astrocytes, microglia, and neurons) can synthesize IL-6 in response to local inflammatory stimuli [46].

Systemic or locally produced cytokines act in the brain to increase the synthesis of cyclooxygenase 2 (COX2), the enzyme responsible for the oxidation of arachidonic acid to produce PGE2, and IL-1 receptors that mediate COX2 induction have been identified in endothelial cells of the brain within the region of the medial preoptic nucleus of the hypothalamus. Although the specific cell types that regulate increased expression of COX2 in response to IL-6 have yet to be identified, blood vessels in the brain express the α subunit of the IL-6 receptor (IL-6Rα), which, together with IL-6Rβ, it forms the functional IL-6 receptor [42].

During the febrile response, the possibility of the involvement of other cytokines is suggested, where, similar to IL-6, the activator of the NF-κB ligand cytokine receptor (RANKL) converges on the COX2 pathway EP3-PGE2, leading to fever induction in the LPS-induced inflammation model. RANKL is best known as a regulator of bone remodeling and lymph-node organogenesis. However, the mRNA encoding RANKL is also produced in the lateral septal nucleus of the brain that interconnects with the hypothalamus, and the RANKL receptor is found in astrocytes in the hypothalamic preoptic region. Although the possible interaction between IL-6 and RANKL–RANK during fever has not been explored, RANKL is proposed as a subsequent mediator of IL-6-induced pyrogenesis [42].

POA neurons project directly into the dorsomedial hypothalamus (DMH) and the rostral medullary raphe region, including the nucleus of the rostral pale raphe and raphe magnus, which receives monosynaptic excitatory inputs from DMH [45].

#### Return to Homeostasis

The immune response during the inflammatory process must be strictly regulated to avoid excessive tissue damage after infection. Furthermore, it makes sense that the effects of feverish temperatures on the immune system are also temporarily regulated during the resolution phase of inflammation; however, the involvement of the underlying mechanisms is not yet fully understood. An example is a rapid restoration of lymphocyte trafficking in high endothelial venules (HEVs) to basal levels within 6 h of interruption of hyperthermia in the fever range [47]. HEV normalization is mediated by zinc-dependent metalloproteinases that cleave endothelial ICAM1 while avoiding other trafficking molecules (such as PNAD); however, it is unknown whether heat stimulates these enzymes’ catalytic activity [42].

Although feverish temperatures initially increase pro-inflammatory cytokine production by macrophages at sites of inflammation [48], there is also evidence that heat stress dampens cytokine synthesis once macrophages are activated. This sequence of events is analogous to natural fever, which often occurs after macrophages and other immune cells initially encounter PAMP. In this sense, macrophages derived from monocytes with an activated phenotype produce less IL-1β, IL-6, and TNF when exposed to febrile temperatures than cells without heat [48]. Heat reduces the transcription of pro-inflammatory cytokines through repressive activities of HSF1, decreased recruitment of NF-κB to the promoter regions of genes encoding cytokines, and decreases cytokine mRNA’s stability. Heat treatment of LPS-activated macrophages also decreases inflammation by inhibiting the release of the inflammatory protein B1 of the high-mobility group DAMP (HMGB1), a ligand for TLR2 and TLR4. Inhibition of HMGB1 release prevents subsequent activation of NF-κB, which controls the synthesis of pro-inflammatory cytokines in immune cells. Strategic temperature changes contribute to a negative biochemical feedback loop that protects tissues against damage from excessive cytokine release after infection [42].

### 3.2. Metabolic Mechanisms of Thermoregulation in the Febrile Response at the Central and Peripheral Levels and Cellular Events

In response to cold or heat, the brain triggers a series of thermoregulatory responses to coping with changes in body temperature. These responses include autonomic effectors, such as thermogenesis, vasodilation, and sweating, as well as behavioral mechanisms that trigger flexible, goal-oriented actions, such as seeking heat or cold, nest building, postural extension [49].

#### 3.2.1. Thermogenesis in Brown Adipose Tissue (BAT)

The brown adipose tissue, specialized in thermogenesis without tremors, produces heat through mitochondrial uncoupling [6]. White adipose tissue (WAT) is responsible for storing energy in the form of triglycerides. Beige adipose tissue has recently been described and involved in heat production through thermogenesis without tremors. This other type of thermogenic fat, the “brown” adipocytes, emerges in deposits located in white adipose tissue (WAT) under some stimuli and possess thermogenic properties [50]. Thermogenesis in BAT and browning in WAT are activated with exposure to cold and sympathetic stimulation with the participation of the hypothalamus as a control center for energy balance, the autonomic nervous system (ANS), and the sympathetic fibers that connect and regulate the various fat deposits.

The hypothalamus controls energy balance through the ANS. Thermogenesis in brown and beige adipose tissues can be induced by activating the sympathetic nervous system (SNS). The control of ANS by the hypothalamus is complex, involving several neuronal populations and signaling pathways in various nuclei. For a profound description of the neuronal populations and signaling paths, consulting Contreras et al. [50] is suggested.

#### 3.2.2. Thermogenesis in Musculoskeletal Tissue (Shivering)

The tremor or chill is the rapid and repetitive skeletal muscle contraction to generate heat [49]. This involuntary thermoregulatory response is driven by a central neuronal mechanism activated by physiological stimuli to increase thermogenesis, such as exposure to a cold environment or the brain’s reception of pyrogenic immune signals during infection or inflammation [51].

The regulation of tremor involves a similar set of structures to those that regulate other physiological responses, including LPB, POA, DMH, and rRPA [49].

POA neurons play critical roles in receiving and integrating information about peripheral (skin and visceral) and local temperatures in the brain and providing appropriate command signals to peripheral thermoregulatory effectors [51]. Thermosensory pathways from skin thermoreceptors to POA have been identified that mediate the forward signaling required to elicit rapid thermoregulatory responses, including shivering, to changes in environmental temperature [6]. Separate pathways transmit cool and warm sensory signals from the skin through the spinal dorsal horn and parabrachial nucleus lateral to the midline portion of the POA, including the median preoptic nucleus (MnPO) [51].

Studies of POA efferent pathways that mediate non-trembling thermogenesis evoked by skin cooling and pyrogen in BAT support the hypothesis that descending GABAergic projection neurons in medial POA (MPO) provide inhibitory regulation of activity of BAT sympathetic neurons in DMH. In infection, immune signaling results in the production of PGE2 in the cerebral vasculature, a pyrogenic mediator, which can act through the EP3 subtype of PGE receptors in the POA to potentially attenuate the activity of downstream GABAergic projection neurons. The cooling- or pyrogen-triggered attenuation of the tonic downward inhibition of POA disinhibits the BAT sympathetic/excitatory neurons in the DMH and the sympathetic premotor neurons in the rostral medullary raphe region, including rRPA, which provides the excitatory impulse for the flow of sympathetic output that determines thermogenesis in BAT [51].

The results of Nakamura and Morrison in 2011 [51] suggest that thermoregulatory and febrile activations of thermogenesis in BAT and thermogenesis with tremors are mediated by altered neuronal discharge in the same regions of the hypothalamus and medulla oblongata.

**Figure 3:**
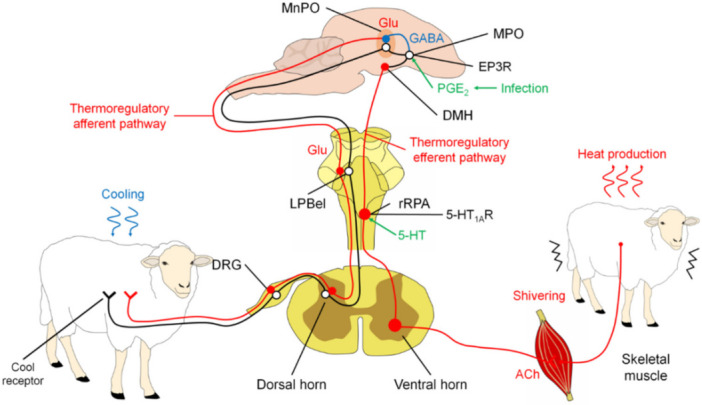
Mechanism of thermogenesis of skeletal muscle. In a cold environment (right), the skin’s cold receptors are activated, and cold sensory signals ascend to the POA through the dorsal horn and the outer lateral part of the lateral parabrachial nucleus (LPBel) and activate GABA neurons in the MnPO, and they then inhibit the GABAergic projection neurons in the MPO. The resulting disinhibition of DMH neurons leads to the activation of neurons in the rRPa, which eventually activates the somatomotor output of the ventral horn neurons, causing shivering. In the case of infection, PGE2, produced in the cerebral vasculature, attenuates the tonic activity of the GABAergic projection neurons in the MPO through the EP3 receptors and, therefore, disinhibits the DMH neurons that drive the output of febrile tremors through the rRPa and the ventral horn. Entry of serotonin into rRPa can inhibit premotor neuron activity through 5-HT1A receptors. Solid red, solid blue, and open black circles denote cell bodies of activated excitatory neurons, activated inhibitory neurons, and suppressed or resting neurons, respectively. Abbreviations: 5-HT1A receptor (5-HT1AR), acetylcholine (ACh), dorsomedial hypothalamus (DMH), dorsal root ganglion (DRG), glutamate (Glu), EP3 receptor (EP3R), external lateral parabrachial nucleus (LPBel), mediate preoptic nucleus (MnPO), medial preoptic area (MPO), prostaglandin E2 (PGE2), preoptic area (POA), and rostral raphe pallidus (rRPA).

The precise pathway that leads to motor neuron activation is unknown. However, rRPA and adjacent structures have been shown to provide polysynaptic input into skeletal muscle, suggesting that they may serve as a common outlet [49] (Figure 3).

#### 3.2.3. Responses in Cutaneous Microcirculation

**Figure 4:**
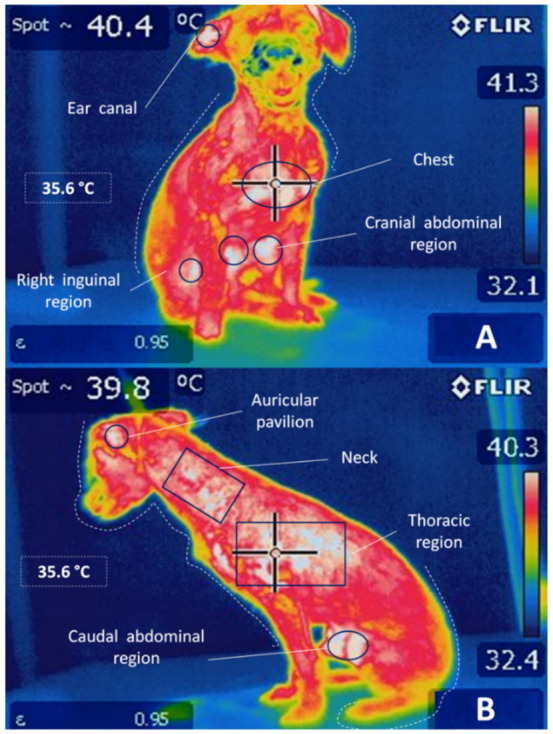
Canine thermograms in a resting state with increased surface body temperatures above normal. (**A**) (above) Temperatures above 41 °C (above) are distinguished in the ear canal, chest, cranial abdominal region, and right inguinal region marked with circles. (**B**) (below) In this thermographic image seen from the side, temperatures above 40 °C are appreciated, marked with circles in the pinna and the caudal abdominal region and rectangles in the thoracic and neck regions. The fact that the thoracic and abdominal areas have higher temperatures than the rest of the body surface indicates that a vasodilation response occurs in these regions that seek to dissipate excess body heat. In contrast, it can be seen that the entire periphery of the body surface (in yellow), marked with discontinuous white lines, shows a reduction in temperature by 5 to 6 °C, compared to the areas marked in circles or rectangles of both thermograms. This decrease in temperature in the periphery indicates that the canine presents a process of peripheral vasoconstriction, characteristic of the onset of the febrile process, to increase the core body temperature and thus increase innate immunity for the destruction of the pathogen that caused the pyrexia. Images obtained with an FLIR E 50 camera equipped with an 18 mm FOL lens at a resolution of 240 × 180 pixels, (emissivity = 0.95, distance = 0.5 m).

The skin acts as a protective barrier between the body and the external environment and functions as a variable capacity heat exchanger. Depending on the temperature detected, a vasomotor adjustment will be carried out in the cutaneous vascular bed to thermoregulate the organism [6,37,52,53]. On the one hand, when faced with cold or fever, cutaneous vasoconstriction occurs to reduce blood flow to the skin and conserve heat in the center of the body (Figure 4) [52,54,55,56]. Alternatively, as a defense response against heat, cutaneous vasodilation increases blood flow to the skin to dissipate heat to the outside [49,57].

The physiological mechanism involved in the development of any of these responses begins with the activation of afferent nerve endings present in the skin, viscera, spinal cord, and brain [58] that detect changes in temperature (thermoreceptors) and send that information to the dorsal horn of the spinal cord. There, the impulse activates warm/cold-sensitive sensory neurons, which, in turn, transmit the nerve impulse to the lateral parabrachial nucleus (LPB), more specifically to the external lateral nucleus (LPBel) in the case of cold signals, or to the dorsal region in the case of warm signals [49]. The LPB sends a glutamatergic projection to the POA, a structure considered the regulatory center of cutaneous vasomotor responses, since the inhibition or cooling of this region induces vasoconstriction, while its excitation or heating induces vasodilation [59]. Once the POA is activated through the efferent pathways described below, vasoconstriction and vasodilation responses occur [60].

In the case of vasoconstriction, a process involved in the fever that reduces heat dissipation to the outside [52], scientific studies suggest that the process begins with the inhibition of neurons of the median preoptic nucleus (MnPO) [61], which appears to stimulate sympathetic premotor neurons for cutaneous vasoconstriction present in the pale raphe pallidus area (RPA) [62,63]. These neurons transmit glutamatergic and serotonergic projections that activate preganglionic neurons in the intermediolateral cell column of the spinal cord, which sends descending projections to the sympathetic ganglions [36]. Finally, these projections reach the efferent fibers that innervate most of the arteries, arterioles, and veins of the body, and cutaneous vasoconstriction occurs [49].

It is worth mentioning that the participation of the ventral tegmental area and the rostroventrolateral PAG in this efferent circuit (downstream) has been proposed [52]; however, it is not yet known how these two regions are functionally connected with POA and rRPA [49].

It is important to highlight that fever can provide protection, since the increase in temperature has been associated with increased innate immunity to bacterial destruction [64]. However, temperatures above 40 °C increase the risk of mortality, so responses are initiated in the body to recover from the fever, such as capillary dilation [16,65].

The neural circuit involved in vasodilation begins when the POA senses the heat signals. Contrary to that described in vasoconstriction, the glutamatergic interneurons present in the MnPO are activated, and they send an excitatory signal to the GABAergic projection neurons of the MnPO and MPA to send an inhibitory stimulus to the raphe pallidus area (RPA) and the rostral ventrolateral medulla, to block the activity of the sympathetic premotor neurons of cutaneous vasoconstriction (CVC) [52]. Then a nerve impulse is transmitted to the preganglionic neurons of the IML, so that it reaches the efferent sympathetic fibers that innervate the cutaneous blood vessels and produce peripheral vasodilation [60].

Regarding the type of activated cell, it is unknown whether, as in the response against cold, glutamatergic cells are responsible for inducing heat-defensive responses, because some studies describe the participation of GABAergic cells [66]. However, shared participation is not ruled out, since POA neurons express both receptors [49].

It is worth mentioning that some species have specialized thermoregulatory organs that allow heat to dissipate more quickly. Examples of them in mammals are the rat tail and the rabbit ears. Both areas of bare skin have an extensive network of blood vessels responsible for the rapid exchange of heat with the environment when the vasodilation process begins [49]. In the case of birds, the bill stands out, because, given its high vascularity, it functions as a thermal window for heat exchange with the environment [67,68,69,70]. Scientific studies indicate that the size of the avian bill influences the rate of heat loss, since species with long bills, such as *Ramphastos toco*, can lose up to 60% of the heat through this structure [70], while in species with small bills, such as *Melospiza melodia*, the total loss of body heat through its surface can represent up to 10% [67,71].

#### 3.2.4. Sudomotor Response

Sweating, in addition to functioning as a means of the excretion of substances such as urea and lactic acid [72], and having chemical substances that confer an immune defense role on the skin (antimicrobial peptides IgA and IgG), belongs to the thermoregulation mechanisms associated with the evaporative pathway [73]. It has the primary physiological function of dissipating heat through the production of sweat, a hypotonic, odorless, and clear solution composed mainly of water, NaCl, and a multitude of solutes in different concentrations [73,74].

Sweat is produced mainly by the eccrine sweat glands distributed in different parts of the body [73]. Its location varies with each species. In cattle, the eccrine sweat glands are distributed on the nasolabial surface, while, in equines, they are more extended in the neck and ventral area [75,76]. In contrast, histological studies do not report the presence of sweat glands in birds, thus leading to the assumption that heat-dissipation mechanisms are more effective in mammals than in birds [58]. However, it has been described that some birds, such as *Columbina inca*, can dissipate heat through a compensatory mechanism known as cloacal evaporation in a percentage very similar to that represented by buccopharyngeal evaporation (21.2% cloacal and 25.4% oropharyngeal). This topic is addressed later in the paper, leading to the conclusion that it is a critical thermoregulatory mechanism in birds; however, it is not yet fully understood how it works [77].

The mechanism’s principle is based on the transfer of body heat to the water found on the s skin’s surface, which subsequently evaporates [73,78,79]. As with other heat-defensive responses, the sudomotor response begins upon detection of the increase in body temperature via thermoreceptors in the POA [80]. Once the POA is activated, it transmits an impulse that stimulates the premotor neurons for sweating, located in the rostral ventromedial medulla (RVMM). These premotor cells project their stimuli towards the preganglionic neurons present in the intermediolateral cell column of the spinal cord, to later be led to sympathetic ganglions that finally innervate the peripheral sweat glands, where acetylcholine is released [49,81,82].

Although the specific circuit that connects the POA with the RVMM is not yet fully understood [49], it is known that the physiological mechanism begins with the binding of acetylcholine from the sympathetic stimulus that they emit non-myelinated sympathetic postganglionic fibers of class C to the muscarinic receptors present in the basolateral membrane of the clear cells, located in the secretory coil of the eccrine glands [73,83,84,85]. This binding triggers the release of intracellular calcium stores and an influx of extracellular calcium into the cytoplasm of clear cells. A cellular shrinkage is caused by the efflux of KCl through Cl channels in the apical membrane and K channels in the basolateral membrane, triggering an influx of Na, K, and Cl through Na-K-2Cl cotransporters in the basolateral membrane and, subsequently, an efflux of Na and K through Na-K-ATPase and K channels in the basolateral membrane, as well as the outflow of Cl through Cl channels in the apical membrane [73,83,84]. Increasing the concentration of Cl in the lumen creates an electrochemical gradient for the movement of Na through the cell junction, while the net efflux of KCl from the cell creates an osmotic gradient for the movement of water into the lumen, through aquaporin-5 channels [73,86,87,88].

Even though sweating proves to be an efficient mechanism for the dissipation of body heat excess, it should be considered that this response can lead to the development of deficiencies of sodium chloride and some trace minerals, such as Ca and Fe, which, depending on their severity, can be fatal [73,89,90,91].

### 3.3. Behavioral Mechanism of Thermoregulation during Fever

Similarly, animals use voluntary behavioral mechanisms to alter the rate of heat loss or absorption, without compromising the use of valuable body resources [49,53,60]. Parallel to autonomic responses, the neurophysiological mechanism begins with detecting a temperature change through thermoreceptors that transmit an input signal that passes through the spinal cord and the lateral parabrachial nucleus (LPB). It then reaches the POA, where it appears that DMH triggers thermoregulatory behaviors [92]. However, there is still much to understand about the neural circuitry involved in the behavioral responses to fever. Starting with the participation of the POA, despite verifying that behaviors such as panting, thermal salivation, and the extension of the body or prone extension can be elicited by applying local heat to the anterior hypothalamus [80,93,94], it has been observed that behaviors such as grooming are elicited by heating the posterior hypothalamus [93]. In addition, in various studies of the effect of injuries on POA, it has been reported that most of the thermoregulatory behaviors remain intact [92,95,96,97,98].

The abovementioned has led to the belief that this structure is not involved in developing these behaviors. This argument is not entirely accepted given the evidence that POA stimulation is sufficient to orchestrate a great variety of thermoregulatory behaviors [49].

Faced with febrile processes, both mammals and birds present behaviors such as loss of appetite, social isolation, and, in more severe conditions, reduced activity, mainly due to the effect of cytokines [7,99]. The development of these behaviors that facilitate the detection of sick animals allows them to save energy in the short-term, reduce the transmission of infection, and ensure the efficient functioning of NK cells and interleukin 2 to respond to the antigen challenge [7,100,101,102,103].

Within these thermoregulatory behaviors carried out in the face of fever, there are some differences between species [49], so they are discussed below.

#### 3.3.1. Thermoregulatory Behavior in Mammals

In rats, it has been observed that, after the onset of fever, induced with LPS, animals seek warm ambient temperatures, probably to optimize their immune response [104]. Likewise, at the beginning of the febrile response, they present adjustments in their body posture that efficiently produce heat. Examples of this are the ball-like posture and basking. On the one hand, the ball posture, commonly observed in small mammals, such as rabbits, reduces the surface-to-volume ratio to minimize surface contact with air and thus reduce the area of heat dissipation [105]. On the other hand, basking, a behavior that various species use to reduce energy demand, consists of heat transfer to the body after exposure of the skin’s surface to solar radiation [106,107].

Similarly, they can use strategies such as grouping or cuddling, developed by gregarious species to limit energy dissipation [108], and nest-sharing behavior based on the constitution of a microenvironment that protects them from environmental changes; both are efficient ways of reducing the energy costs of thermoregulation [105].

Later, when reaching the maximum temperature during the febrile response, which puts the organism at risk, the animals show cold-seeking behaviors and changes in posture that consist of increasing the relationship between the organism’s surface and the air to promote the exchange of heat through conduction, convection, and radiation [58]. Within these strategies, exposure to the wind, water immersion, the extension of the body or prone position, and standing next to inert surfaces or in the shade are behaviors regularly observed in many species [105]. In cattle, although the provision of shade does not eliminate the impact of high heat surges [109], the radiation is lower and, consequently, the caloric load decreases, positively impacting the animal welfare [110].

It should be mentioned that heat dissipation is dependent on the characteristics of the mammalian fur. For example, with less than 1000 hairs/cm^2^, a wind of 14 km/h can penetrate deep into the coat, while at a higher density of fur, even wind of 32 km/h penetrates it little [105,111,112]. Likewise, the color of the coat influences the thermoregulation mechanism: while a light coloration can reduce heat gain, a dark coloration reduces heat loss, even though the skin is protected against the harmful effects of ultraviolet rays due to its high presence of melanin [113]. Therefore, species with higher hair density and dark coloration exhibit difficulties in dissipating excess body heat, so their main dissipation route is sweat [105,111].

In addition to sweat, mammals such as dogs [114] and sheep [115] use panting as another mechanism for dissipating heat through water loss through evaporation. This mechanism, whose explanation is detailed in the section on thermoregulation in birds, has been associated with an increase (by 48%) in lingual blood flow in dogs [116,117] and has been identified as a particularly efficient way to cool the brain [110].

#### 3.3.2. Thermoregulatory Behavior in Birds

Birds have developed a range of physiological, morphological, and behavioral mechanisms to regulate their body temperature [71]. When poultry face temperatures above the comfort zone (41.2–42.2 °C), they spend more time panting and resting than feeding and walking, trying to reestablish their thermal equilibrium [118].

By not having sweat glands [58], birds dissipate body heat through panting, a process that involves energy expenditure. Panting, or thermal polypnea, consists of an increase in respiratory rate and a decrease in respiratory volume, which is believed to restrict hyperventilation to surfaces of the respiratory system that do not participate in the exchange of gases between blood and air, to reduce the possibility of eliminating excessive amounts of carbon dioxide from the blood [119]. This mechanism removes heat through the evaporation of water present in the respiratory tract’s moist lining [120]. However, panting itself generates body heat and can induce respiratory alkalosis when the bird blows off excess carbon dioxide, which can eventually affect the overall performance of the bird [120].

**Figure 5:**
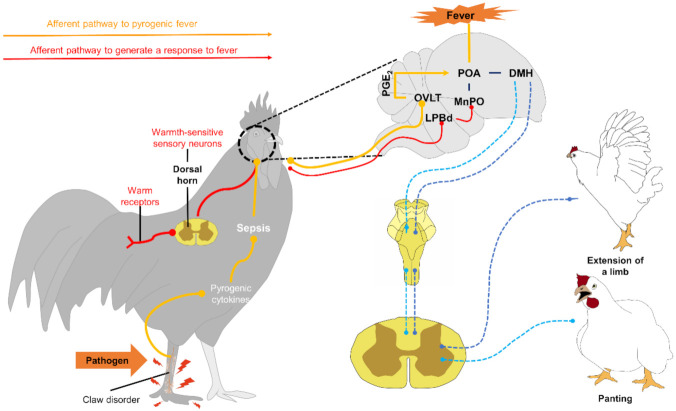
Neurophysiological mechanism for the development of behavioral responses in birds with a febrile process. A hen is shown with a foot lesion that facilitates the entry of a pathogen into the body. The presence of the pathogen leads to the production of pyrogenic cytokines, which, upon reaching the bloodstream, generate sepsis. When these cytokines interact with the organum vasculosum of the lamina terminalis (OVLT), the synthesis of prostanoids, such as PGE2, is stimulated, and they act at the POA level, decreasing the activation speed of heat-sensitive neurons, causing the onset of the febrile process. Subsequently, the thermoreceptors detect the increase in temperature and transmit that signal to the dorsal horn of the spinal cord, where warmth-sensitive sensory neurons are activated. These neurons emit an ascending signal that reaches the lateral parabrachial nucleus of the dorsal subregion (LPBd), which emits a glutamatergic projection to the median preoptic nucleus (MnPO). Once the information is transmitted to the POA, the dorsomedial nucleus of the hypothalamus (DMH) sends an impulse, so that, through still unknown efferent pathways (broken lines), behavioral responses such as panting and extension of a limb are generated to dissipate excess heat.

Regarding body posture, the presence of wings extended away from the body is a typical posture that aims to promote the cooling of the individual by reducing the body insulation and increasing its contact area with the air (see Figure 5). Additionally, they usually splash water on their combs and wattles to increase evaporative cooling on these surfaces [120,121].

They also come to present the behavior of placing the bill within the plumage, since, given its function as a thermal window, doing so could help isolate the bill from environmental heat to prevent it from continuing to gain heat. However, research is still needed to confirm the development of this behavior as a means of insulation against heat, as well as against cold [71].

Despite the fact that the performance of these behaviors generally represents an option to thermoregulate without compromising the energy and water of the body, it must be taken into account that the time allocated to carry them out can have severe consequences on the foraging success and efficiency. This, in turn, can affect the body mass and the condition of the animals [71,122,123]. This is why it is important to resolve situations such as pyrexia on time.

## 4. Importance of IRT in the Detection of Sick Farm Animals

Based on the finding that the use of invasive methods (taking blood samples, rectal temperature, or heart and respiratory rhythm) to evaluate physiological and metabolic parameters in production animals can give unreliable results that reflect the anxiogenic response generated by the procedure itself, infrared thermography (IRT) is used. Contrary to invasive techniques, IRT is a method that does not require too much time and resources [53,124,125,126].

IRT is considered a noninvasive remote sensing method that is useful for evaluating the temperature of animals [53,127,128,129,130] and, more specifically, measure changes in heat transfer and blood flow [126], since variations in thermal patterns are the result of blood flow that intervenes in the amount of radiated heat [131,132].

In veterinary medicine, IRT has been used to assess fertility, metabolism, stress, pain, and health in farm animals [53,126]. Regarding health, thermography has proven to be a helpful method for diagnosing diseases by detecting the hemodynamic changes during the disease process [126,133,134,135]. Next, the findings in various species are described when evaluating different pathological events of clinical importance, using IRT (Table 1).

### 4.1. Coccidiosis

Coccidiosis is considered a highly contagious disease, which commonly affects poultry and rabbits; it is even described as a disease of economic impact, since it has a high mortality rate [136]. It is caused by a protozoan parasite of the genus *Eimeria*, with 15 species. The parasite has an affinity for the gastrointestinal tract, and signs are mediated by the species, time of infection, and the parasite load present in the organism. Signs can range from watery diarrhea in mild cases to watery stools with intestinal mucosa and blood in animals with a chronic course [137,138].

A characteristic sign of *Eimeria* spp. infection is a decrease in temperature until hypothermia. A considerable decrease in temperature has been reported in birds infected with this protozoan before death [139]. It has been mentioned in numerous studies that body temperature is an influential variable to assess the state of health, and that the diagnosis of hypothermia can indicate instability in the body due to the course of some diseases. Therefore, the idea of incorporating the use of IRT arose because it increases objectivity in the diagnosis of diseases such as coccidiosis [140].

Considering that coccidiosis causes a hypothermic state, various studies have been developed using IRT for its diagnosis. They highlight that the method is effective, non-invasive, and allows visualizing variations in skin temperature-specific body regions, detecting changes in the thermal profile [138,141].

#### 4.1.1. Rabbits

In 2010, Vadlejch et al. [138] evaluated the thermal profile of 18 clinically healthy rabbits with ages between 55 and 60 days. The rabbits were divided into three groups: the first two were inoculated with different amounts of *Eimeria intestinalis* oocysts, and the third was used as an infection-free control group. From the inoculation (day zero), the changes in the thermal profile were evaluated utilizing IRT, and daily fecal samples were simultaneously obtained to calculate the oocysts per gram through coproparasitoscopic analysis.

From the tenth day, a significant decrease in rectal temperature, ocular surface, and the auricular pavilion was registered in the group inoculated with the highest amount of *Eimeria intestinalis* oocysts, in contrast to the groups that did not show significant changes. Therefore, they concluded that coccidiosis significantly affects the thermal state of rabbits and thus reaffirmed that the presentation of hypothermia during coccidiosis can be helpful when diagnosing with methods such as IRT.

#### 4.1.2. Birds

Because the body surface temperature in poultry infected with *Eimeria* spp. had not been well investigated, Knížková et al. [139] assessed the changes in the thermal profile of a total of 42 broilers, free of coccidia and raised under controlled conditions. The authors divided the animals into three groups of 14 chickens each: the first two groups were inoculated with different amounts of pure strains of *Eimeria tenella*. Throughout the experiment, daily fecal samples were obtained to estimate the number of oocysts per gram of feces, and from day 8 to 15 after inoculation, thermograms were taken at a horizontal distance of 1 m from each bird. The thermographic images were obtained as quickly as possible to reduce the effects of stress on the birds. Authors measure the variations in specific areas such as the legs and the beak because other studies mention that they are good areas to measure the thermal state of the birds since there is a strong correlation between deep temperature and heat radiation in these areas [142].

The most statistically significant differences were noted 15 days after inoculation, where, as in the previous study, the group that was inoculated with a higher quantity of the purified strain of oocysts showed a drastic decrease of the temperature in areas such as the legs and the bill, compared to the remaining groups. No significant changes were recorded in the ocular and body surface during the experiment. The findings confirm that areas such as the bill and legs are favorable for measuring temperature variations in birds, and the fact that, since coccidiosis is an infection associated with the alteration of thermal homeostasis in birds, IRT is a reliable method when diagnosing this disease [139].

More studies focused on the diagnosis of infections caused by *Eimeria* spp., using IRT are necessary; however, few studies show the usefulness of this tool when evaluating the health status of animals infected with this agent [138].

### 4.2. Mastitis

Mastitis is one of the most discussed animal diseases globally, because it represents severe economic losses for cattle, sheep, and goat producers [143,144]. The invasion of microorganisms causes this disease, generally bacteria, which trigger an inflammatory process in the udder tissue and is classified as subclinical, clinical, and chronic mastitis. The difference lies in the presence of signs of inflammation (redness, heat, swelling, pain, and loss of function). In clinical mastitis, these signs are severe; in subclinical mastitis, these signs are not evident, but changes in the milk composition are detected, while in chronic mastitis, the inflammatory process can last for months [144]. Regardless of the type of mastitis, the dairy industry is affected by reduced milk production (a result of the progressive loss of secretory epithelium) [145,146], premature culling, veterinary expenses, and low-grade milk production [147].

Considering the economic impact of mastitis, the development of methods for its diagnosis is necessary, such as gross examination of the udder and milk color, pH tests, electrical conductivity (EC), California Mastitis Test (CMT), Somatic Cell Count (SCC), biomarkers, and immunoassays [144]. Given these techniques’ subjectivity and laboriousness and the need of methods that can be applied both during milking and the dry period, the study of new alternatives, such as IRT, continues [148].

#### 4.2.1. Bovines

Hovinen et al. [148], to assess the effectiveness of thermography in the early detection of clinical mastitis, used six clinically healthy cows (five Finnish Ayrshire and one Holstein–Friesian), in their first (five cows) or second lactation (one cow), to which 10 μg of lipopolysaccharides (LPS) of *E. coli* diluted in 5 mL of NaCl was inoculated in the left front quarter of the udder to induce mastitis. Throughout the experimental period (5 days), they took thermographic images of the lateral and medial angle of the quarters before carrying out a milk sampling for the analysis of EC, SCC, and N-acetyl-β-D-glucosaminidase activity (NAGase) and a clinical examination. The clinical examination consisted of rectal temperature, the cow’s systemic signs (normal if the cow was alert and had a good appetite, mild to moderate if the appetite decreased and the cow was depressed, and severe if the cow was listless and anorexic). It also included local udder signs (normal if the udder was not swollen, mild to moderate if the udder was slightly swollen, and severe if the udder was sore and very swollen). The appearance of the milk was also estimated (normal if the milk was white and homogeneous; slightly to moderately changed if the milk was slightly discolored or contained small scales; and severe changes if the milk was very discolored, watery, or contained large clots).

The results showed that all cows developed clinical mastitis after challenge with LPS, and that all presented mild to moderate systemic and local signs and changes in the appearance of milk 2 h after challenge. At 4 h post-challenge, an increase of 1–1.5 °C in the quarters’ surface temperature was detected, correlated with rectal temperature, and a rapid decrease until reaching normal levels. Finally, an increase in SCC and EC results was observed, from 4 h post-challenge and in NAGase activity, from 8 h post-challenge, compared to the right frontal quarter that acted as a control group.

**Figure 6:**
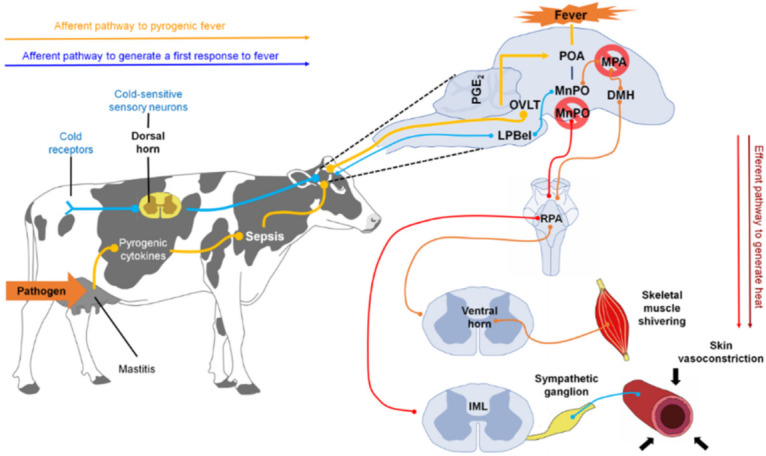
Neurophysiological mechanism for the development of a first physiological response to the development of fever in a cow. The febrile process is triggered by the entry of a pathogen into the body, which releases pyrogenic cytokines that, when interacting with the organum vasculosum of the lamina terminalis (OVLT), increases the synthesis of PGE2. This prostanoid acts at the POA level, causing the development of pyrexia. When the set point of the body temperature is elevated, thermoreceptors detect a low body temperature. They send a nerve impulse that reaches the lateral parabrachial nucleus of the lateral external subregion and is then projected to the median preoptic nucleus (MnPO). Once the information is received, the tremor response is developed by activating the thermogenesis-promoting neurons present in the dorsomedial hypothalamic nucleus (DMH) after inhibition of the medial preoptic area (MPA), which transmit an impulse that passes through the area of the raphe pallidus area (RPA) and the ventral horn of the spinal cord, up to the skeletal muscle. As well as peripheral vasoconstriction, through the activation of vasoconstrictor sympathetic premotor neurons present in the RPA, after inhibiting MnPO activity, which sends glutamatergic and serotonergic projections to the intermediolateral column (IML) to be transmitted to the sympathetic efferent fibers that innervate the cutaneous blood vessels.

**Figure 7:**
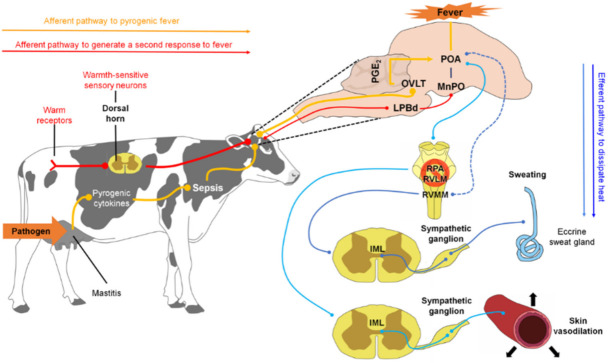
Neurophysiological mechanism of thermoregulation in a cow facing a febrile process. When a life-threatening temperature is reached, thermoreceptors transmit a nerve impulse that reaches the median preoptic nucleus. Once the POA is activated, responses are developed through efferent pathways to dissipate excess heat, such as sweating, which originates after transmitting a nerve impulse to the rostral ventromedial medulla (RVMM) through an unknown pathway (dashed line), and the IML to later reach the sympathetic fibers that innervate the eccrine sweat glands. Moreover, cutaneous vasodilation originates after the emission of an inhibitory impulse to the RPA and RVLM, structures involved in the vasoconstriction process, so that a signal is subsequently transmitted to the efferent sympathetic fibers innervating the peripheral blood vessels.

Unexpectedly the authors observed that the thermal changes in the udder appeared 2 h after the appearance of systemic and local signs and some changes in the appearance of the milk, since, during mastitis, the blood vessels dilate to bring blood cells to the site of infection, and the hyperemia generated results in heat and redness [149]. However, they mention the possibility that this may be due to the edema formation, as the permeability of the capillaries increases and plasma leaks into the interstitium, resulting, in turn, in impaired blood circulation and diminished udder temperature. Another possible explanation is based on the systemic effect generated by the LPS challenge, since they act as an exogenous pyrogen that, through the production of cytokines in the mammary gland, raises the hypothalamus set-point body temperature, causing the development of peripheral vasoconstrictor responses and tremor to try to reach the new temperature, leading to fever [150] (see Figure 6 and Figure 7). This last argument is supported by the increase in surface temperature in the experimental and control quarters, thus reflecting a systemic effect of the LPS. Likewise, it coincides with what was observed by Loughmiller et al. [151], who suggested that detection of the early stage of fever in pigs inoculated with *Actinobacillus pleuropneumoniae* with IRT could not be carried out due to vasoconstriction of the blood vessels.

The authors conclude that the thermal imaging camera is a valuable tool to detect changes in the surface temperature of the udder during clinical mastitis, before the results of milk sampling, but after the development of local signs of mastitis. Therefore, thermography can be used to detect mastitis, at least the clinical one, with fever or other febrile diseases.

Similar results were obtained by Metzner et al. [152], who induced mastitis in five healthy Holstein cows after administering 500 colony-forming units of *E. coli*, strain 1303, in 2 mL of sterile saline solution in the right hind quarter, to determine the changes in the surface temperature of the skin cow udder by taking thermograms of both hindquarters (left hindquarters as the control group), every 2 h, for 24 h before and 24 h after inoculation. Similar to the previous study, all cows developed clinical mastitis and had a swollen right hind quarter, while the left hind quarter showed no clinical signs of mastitis. However, a significant increase in the surface temperature of both quarters was detected between 11 and 17 h post-inoculation, where the maximum temperature of the left quarter reached 40.3 °C, and the right quarter showed 39.9 °C at 13 h post-inoculation, which was when the temperature peak was reached, indicating that there is an uneven distribution of heat during the acute phase of mastitis.

Given the mixed model analysis results, the authors conclude that IRT helps detect *E. coli*–induced mastitis by evaluating the maximum temperature of one or both hind quarters, as long as the intervals between examinations do not exceed 2 h.

Even so, Hovinen et al. [148] point out the need to develop studies that analyze the ability of IRT to detect mastitis under natural conditions, where the systemic inflammatory response may be less pronounced and, consequently, changes in surface temperature can be detected. Such was the case of the study carried out by Polat et al. [153], who used 62 lactating Brown Swiss dairy cows to evaluate the ability of IRT to detect subclinical mastitis compared to the California Mastitis Test (CMT). For this, thermograms of the surface of each quarter and milk samples were taken, also to perform CMT and SCC techniques to determine whether the quarters were healthy (≤400,000 cells/mL) or presented subclinical mastitis (>400,000 cells/mL). The detection of a positive correlation of the thermographic data with the SCC (r = 0.73) and the CMT score (r = 0.86) stands out, and a high percentage of sensitivity and specificity (95.6 and 93.6%, respectively) similar to that of CMT. In addition, it was observed that the quarters that presented subclinical mastitis had a surface temperature of 2.35 °C higher than that in healthy quarters.

Based on results, Polat et al. [153] conclude that thermography is a non-invasive and fast tool and is sensitive enough to detect superficial thermal changes of the udder due to subclinical mastitis, which would be very useful for veterinarians and producers.

#### 4.2.2. Sheep

Martins et al. [143], considering that 95% of mastitis cases in sheep are due to subclinical mastitis, whose detection and treatment is difficult due to the absence of clinical signs, developed a study to assess whether IRT can be used as a tool to diagnose mastitis in ewes. They used 37 Santa Inês ewes between 2 and 5 years old, and they evaluated these ewes weekly, during their lactation (approximately 12 weeks), excluding the perinatal period. This evaluation consisted of taking thermograms of the udder to obtain the temperature of the teats, the front, the intermediate, and rear position of the udder, and later, the milk samples to submit them to the California Mastitis Test (CMT) and the Somatic Cell Count (SCC).

According to the weekly results of the clinical and milk analysis, the sheep were classified into three groups. The first group (healthy) consisted of sheep without tender mammary lymph nodes or udder rigidity, with negative CMT and SCC below 250,000 cells/mL (42% of observations). The second group (subclinical mastitis) consisted of sheep with tender mammary lymph nodes, mild udder rigidity, slightly positive CMT, and an SCC of 250,000–500,000 cells/mL (23% of observations). The third group (clinical mastitis) represented sheep with sensitivity or response in mammary lymph nodes, rigid udders, positive CMT, and an SCC above 500,000 cells/mL (35% of the observations).

The results showed that the subclinical mastitis group presented higher temperatures (36.3 °C) than in the clinical mastitis groups (35.89 °C) and healthy sheep (36.06 °C). Results were similar to those of Hovinen et al. [148], where authors detected a lower temperature in the udder of cows with clinical mastitis. This aspect could be explained by the presence of a chronic inflammatory process, since, as mentioned above, in a chronic stage, the development of edema decreases the blood flow and, consequently, the surface temperature of the organ [150].

Considering that it was possible to correctly classify the mastitis status of the animals with thermographic data (73%), it is concluded that, unlike when milk components are used, infrared thermography is helpful to differentiate the subclinical mastitis group, so that it can be a reliable auxiliary diagnostic method for mastitis in sheep.

In summary, scientific studies have shown that IRT helps detect clinical and subclinical mastitis, both in cattle and sheep, and shows sensitivity and specificity similar to that of methods related to milking, which could not be carried out during the dry period. Although, at least in clinical mastitis, changes in udder temperature are detected after observing systemic and local signs of mastitis [148]. However, the early detection of mastitis through this tool represents the possibility of reducing the biological and economic losses associated with mastitis after executing effective treatments on time [153]. However, research is still needed to evaluate the reliability of this tool under various environmental conditions (temperature, speed, and humidity) and characteristics of the individuals (parity and milk production), which have generally not been considered during experimentation [153], and define how the area to be evaluated should be delimited [152].

### 4.3. Foot Problems

Claw disorders and lameness are among the most critical and complex to treat problems in production systems since the vast majority are not easily visible because they are subclinical [154,155]. These lesions can have different origins, among which, in this paper, we highlight the infectious ones. Infectious lesions originate when a microorganism (bacteria, viruses, and parasites) takes advantage of the presence of lesions to enter and infect deeper tissues of the hoof [156].

Alternative methods or tools are required to detect claw disorders in their early stages [157,158] in order to reduce the incidence of foot problems and the significant negative impact on animal welfare and the finances of the production system [144], because of the pain they generate and the possibility of a spread of the disease [157]. For this reason, studies have been carried out evaluating the use of infrared thermography for the detection of foot lesions in different species.

#### 4.3.1. Bovines

Foot-and-mouth disease (FMD) is one of the viral diseases that most affect the animal trade. The virus is responsible for the appearance of vesicles on feet, mouth, tongue, and nipples in cloven-hoofed animals, and it is one of the most contagious pathogens [159].

Despite being a disease eradicated in many regions of the world, there have been cases of reintroduction with devastating economic, social, and environmental effects [160], so countries must demonstrate freedom from the disease and its causal agent in their animal population. Considering that the performance of clinical examinations is time-consuming and may not be sufficient for the detection of infected animals without obvious clinical signs, Rainwater-Lovett et al. [159] carried out a study to evaluate the use of IRT as a detection method for cattle infected with the foot-and-mouth disease virus (FMDV) and its possible application in the identification of suspicious animals and the confirmation of diagnoses during some outbreak. They used 6-to-8-month-old Holsteins steers, weighing 180–270 kg, classified into three groups: DI, inoculated directly with a virus suspension (*n* = 12); CT, animals in contact with steers of the DI group (*n* = 6); and VT, animals that were part of a vaccine trial and that were also inoculated directly with the virus (*n* = 21). Thermographic images were taken before and after inoculation or contact with infected animals, daily, to capture three stages of infection: preclinical (one day before identifying foot lesions, clinical (the first day that foot lesions were identified), and post-clinical (one day after the foot lesions were identified). Each leg temperature was evaluated from the bottom of the hoof to the top of the toes. After taking thermograms, the steers were sedated to visually examine vesicular lesions on the legs, nose, mouth, and tongue and score them with a numerical scoring system. The infection status was established by clinical evaluation and laboratory confirmation.

The authors performed an analysis to identify the best site for detecting FMD where they compared the maximum temperature of the legs, the face, and the rectum with the clinical scores. Despite finding moderate positive correlations between the maximum temperature of the legs and the rectal and ocular temperature (r = 0.53 and r = 0.60, respectively), the results of the analysis showed that the legs were the best window, because the temperatures of this site were parallel to clinical scores in 82.4% of unprotected cattle. Eye temperature is generally used because it is believed to reflect internal body temperature [141]; however, the study did not show a strong positive correlation between facial and rectal temperature (0.50), so Rainwater-Lovett et al. [159] used the legs’ temperature, which increased consistently before the development of fever.

Among the findings, the detection of similar increases in the leg temperature of the bovines that made up the DI and TC group, regardless of the viral strain or the route of exposure to FMDV, stands out. After FMDV inoculation, DI steers showed an increase of 4.7 °C during the preclinical stage, compared to the baseline average of healthy bovines, and 7.2 °C in the clinical post-clinical stages. In the case of CT steers, increases of 4.8, 7.5, and 8.9 °C were detected in the preclinical, clinical, and post-clinical stages of the infection, respectively.

On the contrary, the bovines of the VT group presented lower temperature increases (0.5 °C in the preclinical stage, 5.7 °C in the clinical stage, and 5.2 °C in the post-clinical stage), which could reflect a lower inflammatory response, a product of the partial protection of the vaccine, because 38.1% developed viremia and foot lesions.

In the study, 50% of the animals in the CT group were detected as positive for FMD, using the IRT, one day before having detectable viremia and two days before developing foot lesions. One day after exposure, 58.3% of DI steers tested positive for FMD through IRT, and viremia was detected that day. It should be noted that, at least in the bovines of these groups, the clinical signs never arose before the viremia.

Given the ability of IRT to detect animals infected with FMDV by contact, not only in the preclinical phase, before presenting foot lesions, but also during the pre-viremic phase, it can be concluded that this tool, which is fast and not invasive, may be helpful for the early detection of animals with FMD. It could even be used to improve the selection of animals to be sampled, resulting in a more efficient use of valuable resources and faster quarantine implementation. However, considering that other pathologies trigger fever and cause foot inflammation, it is necessary to complement the use of IRT with traditional diagnostic methods [159]. Since it remains unconfirmed whether the usefulness of IRT can be limited in detecting of FMD in animals that have received vaccines against the virus, it is recommended to continue with research to evaluate if there are variations with other species or other environmental conditions [159].

#### 4.3.2. Horses

Lameness is considered one of the most frequent health problems in horses (*Equus ferus caballus*), causing loss of performance [161,162]. In the pathophysiological process of lameness, the digital circulation is altered, which is essential for the transport of nutrients, waste elimination, and the correct hoof thermoregulation, which, due to its rigid nature, makes the equine foot particularly susceptible to pressure changes, as there is little space for expansion to accommodate the edema that characterizes any inflammatory process [163,164].

Among the most common causes of lameness in horses is navicular syndrome or laminitis [165]. One of the most frequent causes of laminitis in horses is the provision of high-energy diets that are low in fiber [166], because carbohydrate overload alters the bacterial balance of the gastrointestinal tract, causing excessive growth of lactic acid-producing bacteria, which change the pH, usually neutral, to an acidic medium. Acidity causes the death of Gram-negative bacteria of the genus Enterobacteriaceae and the release of LPS, which, in action with acidic pH, damages the mucosa and allows the passage of endotoxins into the portal circulation, causing the release of inflammatory mediators and, subsequently, the decrease in capillary perfusion of the claw, edema, and ischemia [167].

Considering the role that digital circulation plays in lameness and that IRT is a method that allows evaluating the blood flow of various tissues without requiring the approach to a blood vessel as when using Doppler ultrasound, Douthit et al. [165] evaluated the correlation of hoof surface temperature and ultrasound measurements of digital blood vessels to determine whether these measurements can be used as predictors of clinical lameness. They used 12 three-year-old American quarter-mile horses, namely six mares and six geldings, and an average initial weight of 459 ± 31 kg, which had previously been used to evaluate the effect of consumption of endophyte-infected fescue on the digital circulation [168]. The surface temperature of the horse’s front hooves was measured approximately 1 cm below the coronary band, in 15-min intervals, starting 75 min after the morning feeding. On the other hand, Doppler ultrasound was used to measure the diameter and velocity of the blood flow of the medial palmar artery of the left forelimb, starting 60 min after the morning feeding and repeating the measurements every 30 min. Simultaneously, clinical lameness tests were performed.

The statistical results showed that there is a moderate correlation (0.40) within the horse between the surface temperature of the hoof and the speed of blood flow, similar to that reported by other authors [169,170]. Douthit et al. [168] mention that this result can be attributed to measurement noise associated with equipment, operator, or environmental variations; however, the authors made an effort to minimize environmental interference.

In contrast, a correlation of 0.99 was detected, at horse level or between horses, between the hoof surface temperature and the speed of blood flow in the distal limb, leading to the conclusion that the temperature of the hoof can be used to predict the speed of blood flow on a horse level basis. However, no significant associations were found between speed, diameter, volume, or temperature and lameness, although other studies have reported that IRT has successfully diagnosed lameness in horses [171,172]. For this reason, the development of research with a larger sample size is suggested to determine, with adequate statistical power, whether or not these measurements are significantly associated with lameness and its degree.

#### 4.3.3. Birds

Footpad dermatitis (FPD) is a global problem in growing turkeys, affecting animal welfare due to the potential pain involved [173,174].

Moe et al. [174], based on the premise that with IRT, it was possible to identify subclinical infections of the pad (“bumble foot”) in laying hens [175], developed a study where they investigated the variation of the temperature surface of the sole of the paw, within and between individuals, in two plantar subregions (the pad and the entire plantar surface, including the interdigital membranes). Moreover, the effect of cleaning this region, of obtaining more knowledge about the use of thermography in avian medicine and, particularly, for detecting paw health problems. They used eighty 10-week-old male turkeys (*Meleagris gallopovo*), which were held manually to take thermographic images of the surface of the right leg and its pad, before and after being cleaned (with warm water and a sponge, and dried with a paper towel), to assess the severity of the possible FPD visually. The evaluation was carried out through a visual analog scale (VAS) based on a 4-point scale, where 0—no lesions; 1—superficial lesions, and each papilla is still visible; 2—severe lesions with dark crusts covering less than 50% of the pad; and 3—severe lesions with dark-colored crusts that cover more than 50% of the pad.

Among the results, it is highlighted that most of the pads obtained a score of 1, that is, a slight FPD. There was a negative association between the results obtained with the VAS and the IRT temperatures: the larger the areas with discoloration, the lower the temperatures detected on the plantar surface and the pad. This last result contrasts with Wilcox et al. [175], who found that the increase in the severity of pododermatitis in laying hens increased surface temperature. Moe et al. [174] suggest that this discrepancy may be because hens (60 weeks old) presented a more severe inflammation process than turkeys, an argument that is supported by the description of pododermatitis (bumblefoot) lesions (“pustules and swelling visible at first glance”).

On the other hand, the authors speculated that the signs of FPD detected in the study (discoloration spots on the skin surface are associated with the onset of ischemic necrosis, as has been observed in the initial stage of FPD in other species of birds [176], which could explain the low temperatures generated by the reduction of blood circulation of the plantar surface. However, they also consider that the results could indicate the development of hyperkeratosis, a process that, at the cellular level, is observed together with acute inflammation and necrosis of the epidermis [177,178], and that the keratin surface could have protected the emission of heat from possible inflammatory processes in the pad. The above is a situation that would also explain the detection of significantly lower temperatures (−3.36 ± 0.28 °C) in the pad than in the foot since the interdigital membranes of the foot have a thin skin that receives more radiation compared to the thick layers of keratin present in the pad.

These results indicate that IRT is a reliable tool for detecting subclinical foot pathologies in turkeys. However, there is still a need to standardize the protocols when using IRT to study abnormalities in leg health in turkeys, as the anatomical region of interest needs to be precisely defined and the effect of handling time and sequential order of the test, aspects in which Moe et al. [174] detected differences. Similarly, the development of histological studies is recommended to verify the hypotheses raised.

### 4.4. Bovine Respiratory Complex Diseases

Bovine Respiratory Complex Diseases are described as a series of infectious diseases of multifactorial origin caused by various pathogens, mainly viruses and bacteria [179]. The most frequently involved viral agents include the Bovine Infectious Rhinotracheitis virus (RIB), Parainfluenza-3 virus (PI3), Bovine Respiratory Syncytial Virus, and Bovine Viral Diarrhea Virus (BVDV). These are related as predisposing or synergistic factors with bacteria and related to the origin of immunosuppression that allows the development of simultaneous secondary infections caused mainly by agents such as *Mannheimia haemolytica*, *Mycoplasma bovis*, *Pasteurella multocida*, and *Haemophilus somnus* (*Hystophilus sommni*) [180,181]. These agents affect the respiratory tract and colonize others as the digestive and reproductive systems, with different clinical signs observed, of which the most characteristic are dyspnea, cough, fever, nasal discharge, anorexia, and lethargy.

The respiratory complex in intensively raised calves is increasingly common, and as a result, these alterations have a high economic impact due to the increase in mortality rates and the high costs of treatment; due to the frequency of presentation of these diseases, livestock industries are subject to the use of antibiotics. However, concern has been expressed about poor practices in managing of antibiotics that cause a decrease in the capacity to treat these diseases due to increased antimicrobial-resistant strains [182]. In addition, various sources have reported that early diagnosis is necessary to improve the selectivity and effect of antibiotics against etiological agents. However, there are complications in the case of the respiratory complex, since the signs appear when the course of the disease becomes chronic [183,184]. Early detection methods have been sought to diagnose and treat diseases quickly with positive results based on this complication. An example of this is the detection of acute-phase proteins, such as endogenous pyrogens. However, it is considered a complicated method, since the diagnosis cannot be carried out in real-time, due to the necessary collection and subsequent analysis of the samples to determine the presence of these analytes [185].

Another method with potential diagnosis is thermal imaging cameras, so recent studies have focused on determining the reliability of a diagnosis through it. Starting from this premise, Schaefer et al. [186] decided to determine the efficacy of IRT in the early diagnosis of respiratory diseases in cattle. They used 133 weaned calves previously examined to rule out the infection of any agent involved in respiratory diseases and thus avoided the bias of the information, due to differences in the course of the disease. Simultaneous with evaluating the variations in the thermal profile, hematological measurements, physical examinations, estimation of salivary cortisol, body weights, and serological examinations were carried out at weekly intervals to reaffirm the diagnosis.

To determine if the animal was sick, used a criterion that is considered the gold standard that stipulates that an animal that presents two or more of the clinical signs defined in the parameter is considered a genuine or positive disease [187].

In the study, they obtained images of the ocular surface by focusing the camera on the orbital area, which includes the eyeball and part of the skin that surrounds the eye socket, since they started from the argument that this area, together with the lacrimal gland, is favorable for measuring variations in temperature due to its sensitivity to changes in thermoregulation.

The results showed that the clinical scores, hematological values, and thermograms indicated statistically significant similarities when diagnosing the animals. However, it was surprising to discover that the values obtained by IRT were more efficient than the clinical values since the information obtained 4–6 days before the presentation of clinical signs showed that the thermograms were notably more effective for recognizing true positive animals. They even describe this method as one with predictive potential. However, it is concluded that more research is necessary to optimize the reference values of the thermal profile for the early detection of these diseases in conjunction with the optimal method to use these parameters [186].

It is worth mentioning that similar results were obtained by Schaefer et al. [183] in a study whose objective was to investigate the level of reliability of IRT when used as a method for the early detection of systemic diseases, such as Bovine Viral Diarrhea (BVD). They used 15 calves of approximately 172 kg, distributed into three groups of five; the first two groups were inoculated with type 2 virus, and the last group remained seronegative as a control. Throughout the study, they monitored the changes in the thermal profile, focusing on the bovines’ facial surface and conducting complementary studies simultaneously. They emphasized that the detection or prediction of the disease through thermographic images occurred one week before the complementary clinical studies indicated the existence of the course of a disease, so based on these findings, they suggested the development of an early prediction index by using this diagnostic method [183].

**Table 1:** Recent advances related to different pathologies in farm animals through the use of IRT.

Species	Objective	Findings	Implications	Authors
Rabbits	To determine the changes in the thermal profile of rabbits infected with *Eimeria intestinalis* through thermographic images to detect the variations caused by the infection.	The inoculated group with the highest number of oocysts showed a statistically significant decrease in rectal, ocular surface, and auricular pavilion temperatures, in contrast to the remaining groups (inoculated with a lower amount and uninoculated group control).	Since coccidiosis significantly affects the thermal state of animals, it is suggested that IRT can be considered a reliable method to diagnose this disease.	Vadlejch et al. [138]
Horses	To assess the correlation of hoof surface temperature and digital blood vessel ultrasound measurements to determine whether these measurements can be used as predictors of clinical lameness.	There is a 0.99 correlation between the hoof surface temperature and the velocity of blood flow in the distal limb, indicating that the temperature could be used to predict the velocity with which the blood flows from the foot.	Infrared thermography could be used to detect vasodilation and vasoconstriction responses in horses’ feet, which could be associated with lameness and its degree.	Douthit et al. [165]
Bovines	To evaluate the effectiveness of thermography in the early detection of clinical mastitis induced by *E. coli* lipopolysaccharides.	An increase of 1–1.5 °C was detected in the quarters that presented induced mastitis 2 h before the diagnostic tests related to milk.	Thermography can be a valuable tool for rapidly detecting changes in udder surface temperature associated with clinical mastitis, even during the dry period.	Hovinen et al. [155]; Metzner et al. [161]
	To evaluate the subclinical mastitis detection capacity of infrared thermography compared to California Mastitis Test (CMT).	A 2.35 °C increase was detected in quarters with subclinical mastitis than healthy quarters; there is a positive correlation between thermographic data and the CMT score (0.86).	Thermography is sensitive enough to detect superficial thermal changes in the udder caused by subclinical mastitis, a pathology challenging to diagnose.	Polat et al. [153]
	To evaluate the use of IRT as a method of detection of cattle infected with foot-and-mouth disease (FMD).	With the IRT, it was possible to detect the infection of 58.3% of the steers inoculated directly and 50% of the animals that had contact with the first ones, who presented an increase in the temperature of the feet between 4.7 and 8.9 °C, before developing foot injuries.	IRT can be used to detect FMDV-infected animals early, in the preclinical phase (before presenting foot lesions) and during the pre-viremic phase, or those that must be sampled, to speed up the implementation of quarantines.	Rainwater-Lovett et al. [159]
	To determine the efficiency of IRT as a method for the early diagnosis of respiratory diseases in bovines.	Values obtained by the IRT method were more efficient than the clinical values of hematological measurements and physical examinations. The authors even describe that the method has predictive potential when detecting true positives.	Considering that early diagnosis of these diseases is necessary to reduce bad practices with antibiotics, IRT is an efficient, non-invasive method that reflects results in real-time compared to other methods.	Schaefer et al. [186]
Sheep	To assess whether IRT can be used as a diagnostic tool for mastitis in sheep.	With the IRT, it was possible to correctly classify the mastitis status of the animals in 73%, where it was detected that the subclinical mastitis group had a higher temperature (36.3 °C) than in the clinical mastitis groups (35.89 °C) and healthy sheep (36.06 °C).	Unlike when milk components are analyzed, IRT can be used as an auxiliary method to diagnose subclinical and clinical mastitis in sheep.	Martins et al. [143]
Birds	To assess the detection of footpad dermatitis (FPD) through variations in the surface temperature of the footpad and the whole plantar foot surface in turkeys.	It was possible to detect a mild FPD in most turkeys through IRT, which presented lower surface plantar foot temperatures, the larger their area with discoloration, was associated with hyperkeratosis or ischemic necrosis.	IRT is a reliable tool that can be used to detect subclinical pathologies in the turkey foot.	Moe et al. [174]
	Assess the variations in the thermal profile of broilers during *Eimeria tenella* infection and provide ranges or criteria for future evaluations with the IRT method.	At 15 days after inoculation, a statistically significant decrease in temperature was observed in areas such as the shank and the bill surface in the group administered the highest amount of the purified strain.	IRT can be used as an effective method to detect diseases such as coccidiosis, and the areas described as most sensitive to temperature changes in broilers are the shanks and the bill.	Knizkova et al. [139]

## 5. Conclusions

Fever refers to an acute phase response that confers a survival benefit on the body, with the ability to raise core body temperature during processes of infection or systemic inflammation, to reduce the survival and proliferation of infectious pathogens through the alteration of temperature, the restriction of essential nutrients, and the activation of an immune reaction. However, once the infection is resolved, the febrile response must be tightly regulated to avoid excessive tissue damage.

Currently, a considerable portion of the immune, metabolic, and neural pathways and the structures involved both in the development of the febrile response and, in return, to thermal homeostasis when infection or inflammation has ceased are known. There is still much information to be revealed about the underlying mechanisms that participate in the febrile process and the control of energy balance. However, the data obtained to date have been of great value for the more precise identification and diagnosis of certain diseases in production animals and interpreting the results obtained through different tools.

Infrared thermography is a fast, reliable, and non-invasive tool that can be useful in the early detection of pathologies of clinical importance, which affect the well-being and productivity of farm animals. Depending on the pathology and the species under study, increases (as in the case of pad dermatitis) or decreases (as in coccidiosis) in the surface temperature of specific thermal windows (feet/shank, bill, udder, eye region, and auricular pavilion) will be observed.

However, considering that a great variety of pathologies trigger a fever, it is necessary to complement the results of the IRT with basic tests that allow us to confirm the diagnosis. Similarly, it is suggested to continue with research in this area to confirm the results obtained with small samples, evaluate whether the results differ between species, and conclude standardizing the protocols for the use of this tool in order to avoid variations due to the effect of technical factors.

## Data Availability

Not applicable.
